# Do existing research summaries on health systems match immunisation managers' needs in middle- and low-income countries? Analysis of GAVI health systems strengthening support

**DOI:** 10.1186/1471-2458-11-449

**Published:** 2011-06-08

**Authors:** Xavier Bosch-Capblanch, Marion Kelly, Paul Garner

**Affiliations:** 1 Swiss Tropical and Public Health Institute, Socinstrasse 57, Basel 4051, Switzerland; 2 University of Basel, Basel, Switzerland; 3 Liverpool Associates in Tropical Health, 25 Anson Street, Liverpool L3 5NY, UK; 4 Liverpool School of Tropical Medicine, Pembroke Place, Liverpool, L3 5QA, UK

## Abstract

**Background:**

The GAVI Alliance was created in 2000 to increase access to vaccines. More recently, GAVI has supported evidence-based health systems strengthening to overcome barriers to vaccination. Our objectives were: to explore countries' priorities for health systems strengthening; to describe published research summaries for each priority area in relation to their number, quality and relevance; and to describe the use of national data from surveys in identifying barriers to immunisation.

**Methods:**

From 44 health systems strengthening proposals submitted to GAVI in 2007 and 2008, we analysed the topics identified, the coverage of these topics by existing systematic reviews and the use of nation-wide surveys with vaccination data to justify the needs identified in the proposals.

**Results:**

Thirty topics were identified and grouped into three thematic areas: health workforce (10 topics); organisation and management (14); and supply, distribution and maintenance (6). We found 51 potentially relevant systematic reviews, although for the topic that appeared most frequently in the proposals ('Health information systems') no review was identified. Thematic and geographic relevance were generally categorised as "high" in 33 (65%) and 25 (49%) reviews, respectively, but few reviews were categorised as "highly relevant for policy" (7 reviews, 14%). With regard to methodological quality, 14 reviews (27%) were categorised as "high".

The number of topics that were addressed by at least one high quality systematic review was: seven of the 10 topics in the 'health workforce' thematic area; six of the 14 topics in the area of 'organisation and management'; and none of the topics in the thematic area of 'supply, distribution and maintenance'. Only twelve of the 39 countries with available national surveys referred to them in their proposals.

**Conclusion:**

Relevant, high quality research summaries were found for few of the topics identified by managers. Few proposals used national surveys evidence to identify barriers to vaccination. Researchers generating or adapting evidence about health systems need to be more responsive to managers' needs. Use of available evidence from local or national surveys should be strongly encouraged.

## Background

Global health initiatives often focus on a disease (such as TB) or on health outcomes (such as cure rates) to help programmes be effective. However, this can unwittingly overburden already weak health systems[1]. The debate between delivering services through vertical or horizontal mechanisms has endured since the 1960's, with continued calls to integrate programmes and activities into existing services.

The "Expanded Programme on Immunisation" (EPI), which was launched in the late 1970s, has been linked with other interventions (such as growth monitoring, oral rehydration salt solutions and breast feeding promotion) or integrated into wider primary health care services. Vaccination coverage went up initially, but in the late 1990s immunisation coverage stagnated. The GAVI Alliance (GAVI) was set up in the year 2000. Its mission is to save lives by accelerating access to existing underused vaccines, strengthening health and immunisation systems in countries and introducing innovative new immunisation technology, including vaccines[2]. Some critics accused GAVI of being too centred on immunisation at the expense of strengthening health systems[3].

In response to this, GAVI allocated funds to support health systems strengthening, based on a set of principles established through consultation with stakeholders and debate within its Board [4-6]. GAVI opened a support line for countries to address their health systems priorities by submitting proposals based on identified health systems weaknesses and to stimulate the use of research evidence to help inform the ways to address the weaknesses so identified. This support may complement other existing health systems strengthening initiatives in countries. GAVI set up three thematic areas[7] for countries to articulate their proposals:

1. Health workforce mobilization, distribution and motivation, targeted at those engaged in immunisation and other health services at the district level and more peripheral levels;

2. Organization and management of health services at the district level and more peripheral levels (including financial management);

3. Supply, distribution and maintenance systems for drugs, equipment and infrastructure for primary health care.

The GAVI guidelines for these applications to support health systems strengthening state that proposals should be based on recent immunisation and health sector analyses, and show the appropriateness of the health systems strengthening strategies that are proposed to overcome the identified barriers to immunisation[8]. In most cases these proposals were written in-country by a team of government and external development partners with the assistance of a consultant; proposals were then endorsed by key stakeholders relevant to the delivery of vaccines in the country and reviewed by GAVI and an independent review committee before final approval[9].

Despite the growing number of countries, political leaders and international health experts recognising the need to make a major and sustained commitment to strengthening health systems[10], summaries of reliable research in health systems, often termed "evidence" of policy and strategies, is judged by to be scarce[11]. We made use of a unique opportunity to test this by examining managers' expressed needs against the availability and utility of evidence to support health systems strengthening initiatives, using the HSS proposals that GAVI makes available on its website. Our objectives were to:

◇ Explore countries priorities for health systems strengthening;

◇ Describe the number, quality and relevance of published research summaries for each priority area;

◇ Explore the use of nation-wide surveys to identify barriers to immunisation.

## Methods

### (1) Countries' Priorities for Health Systems Strenghtneing

From the 44 proposals submitted in 2007 and 2008, we extracted the topics prioritized by countries for GAVI health systems strengthening funding. Each topic was listed under one of the three GAVI-specified health systems strengthening areas (see introduction)[7]. We used a pre-tested data extraction form and aggregated related topics. We used the number of times a topic appeared in the objectives across all proposals as a proxy to rank the importance of a topic.

### (2) Describe the Existing Research Summaries and Syntheses for Priority Topics

We searched for systematic reviews related to health systems strengthening. We defined our inclusion criteria as: any systematic review, traditional narrative review or overview of reviews that dealt with interventions or strategies related to any of the topics in the list generated from the proposals. We systematically searched the literature using the terms and databases outlined in annex 1, constraining this to reviews published in a 10-year period up to mid-2009. Abstracts of retrieved reviews were assessed against the inclusion criteria.

We tabulated for each review: the type of review; the kinds of studies included; the sites of the included studies; the nature of the intervention(s); and the outcome(s) measures. Included reviews were rated for methodological quality and relevance.

#### Methodological quality

Potential bias ratings for selected reviews were made independently by MK and by PG, taking into account the approach (i.e. systematic review, narrative review), the clarity of the research question, the quality of the search strategy, the explicitness of data extraction methods, the kinds of studies included, the description of studies, the presentation of results and the clarity of links between results and conclusions.

Reviews and overviews were classified as high quality if they used a systematic approach, if the quality of the included studies was assessed and if no major methodological limitations could be identified. Differences in ratings were resolved by discussion. A judgment on the quality of each review was entered in the table of included studies together with a brief rationale for the judgment.

#### Relevance

Three dimensions of relevance were assessed for each review: thematic, geographic and policy.

Thematic relevance was high if reviews specifically addressed any of the three main areas; moderate if topics were addressed as part of a wider theme; and low if topics were marginally addressed without specific evidence provided.

Geographic relevance was high, moderate or low depending on whether the reviews were focused on low- and middle-income countries[12] (LMIC), included evidence from both LMIC and non-LMIC contexts, or provided no evidence specific to LMIC, respectively.

Policy relevance was assessed by looking at the firmness of conclusions reached by the reviewers and the strength of recommendations made by the reviews' authors: high relevance if specific recommendations were produced; moderate relevance if recommendations were unspecific or vague; and low relevance if no policy directions could be drawn from the reviews.

Moderate and low relevance were grouped in the presentation of results.

#### Mapping to theme

Each one of the reviews or overviews was matched against the topics to describe what evidence was available for the topics in each of the three main areas, graded by the frequency in which each topic appeared in health systems strengthening proposals.

### (3) Use of Nation-Wide Surveys

In its guidelines for health systems strengthening proposals, GAVI encourages countries to use local evidence regarding barriers to immunisation to support their proposals. Nationally representative household surveys (e.g. Demographic and Health Surveys[13] -DHS- and Multi-Indicator Cluster Surveys[14] -MICS-) are available from many of the countries eligible for GAVI support and are publicly accessible. To assess the use of these data, countries' proposals were scrutinised for references to nation-wide surveys and compared with the availability of reports of those surveys at the time of proposal writing.

## Results

### (1) Countries' Priorities for Health Systems Strengthening

Of the 44 country proposals, the majority were from Africa (23), followed by Asia (15), and a few from Latin America and the Caribbean (4) and Eastern Europe (2) (Table 1). From the 44, 30 different topics were identified across all proposals and are described in Table 2. Ten were in the health workforce category, 14 concerned organisation and management, and 6 related to supply, distribution and maintenance.

**Table 1:** Countries that submitted health systems strengthening proposals 2007-2008, by region

Africa	Asia
Burkina Faso	Afghanistan
Burundi	Azerbaijan
Cameroon	Bangladesh
Central African Republic	Bhutan
Chad	Cambodia
Congo	Indonesia
Côte d'Ivoire	Korea
Eritrea	Kyrgyzstan
Ethiopia	Myanmar
Ghana	Nepal
Guinea	Pakistan
Kenya	Sri Lanka
Liberia	Tajikistan
Madagascar	Vietnam
Malawi	Yemen
Mali	**Eastern Europe**
Nigeria	Armenia
Rwanda	Georgia
Senegal	**Latin America and Caribbean**
Sierra Leone	Bolivia
Sudan	Cuba
Uganda	Honduras
Zambia	Nicaragua

**Table 2:** Topics identified by country managers as priorities ranked within themes, and the number of reviews identified for each topic

Themes	Managers priorities in proposals		Number of high quality reviews
	N	%	(total)
**1. Health workforce**			

Developing staff skills to deliver PHC services (not necessarily specific to immunisation)	31	70%	4(16)

Strengthening supervision, for example, by developing checklists or providing transport	26	59%	2(8)

Fill vacant posts for specific cadres by training, or incentives for recruitment or retention	18	41%	0(2)

Performance incentives for individuals and teams, creating or implementing rewarding	18	41%	3(7)

Develop hands on skills in delivering immunization	12	27%	1(3)

Strategies to retain staff and ensure equitable geographical distribution	10	23%	1(8)

Developing and introducing staff performance management systems	6	14%	0(1)

Redistributing and delegating tasks across staff (through training, supervision, guidance)	4	9%	5(12)

Measures to increase the number of female staff	3	7%	0(1)

Creating new cadres such as District Health Officers, not specific to immunisation	1	2%	3(6)

**2. Organisation and management**			

Health management information systems, Monitoring and Evaluation, to inform decisions	39	89%	0(3)

Training managers in planning and budgeting	30	68%	0(1)

Increase knowledge, awareness and community empowerment to promote demand	28	64%	8(19)

Outreach delivery of services, mass campaigns; providing transport/logistics, allowances	22	50%	1(2)

Systems for quality control; training; develop QA programmes; assure drug quality	21	48%	0(3)

Skills in financial management; training managers, administrative staff	14	32%	0(0)

Community participation in management, information systems and oversight	14	32%	0(2)

Integrating (vertical) programmes or activities into PHC	10	23%	3(8)

Improving referral (e.g. providing transport), strengthening secondary care services	10	23%	2(3)

Financing, introducing health insurance; economic studies to inform decisions	8	18%	1(7)

Outsourcing PHC services, contracting out service delivery to non-government providers	8	18%	1(10)

Performance contracts within the public sector (e.g. between central and district levels)	6	14%	0(0)

Improving aid effectiveness through sector-wide approaches and creating basket funds	3	7%	0(0)

Oversight within public sector, strengthening District Health Management Teams	2	5%	0(0)

**3. Procurement and supply management**			

Improving storage, transport, procuring vehicles	32	73%	0(1)

Adequacy of health equipment, procuring and distributing equipment for health facilities	24	55%	0(0)

Adequacy of non-health equipment, procuring other equipment (e.g. computers)	22	50%	0(0)

Condition/amenities of health facilities, upgrading health facilities	20	45%	0(0)

Procuring and distribution essential drugs and other key commodities	16	36%	0(1)

Build new facilities in underserved areas	14	32%	0(0)

PHC: Primary Health Care. QA: Quality Assurance.

With regard to **the health work force **theme, the highest demand was for specific staff training and skills development in existing health care delivery programmes, and the strengthening of supervision (Table 2). Following this, priorities included incentives and rewards, either to accept vacant posts, to retain staff in post, or to improve performance.

Within the **organisation and management **theme, managers requested support to improve health information systems and to train managers in budgeting and planning. Other priorities included community-oriented interventions (increasing demand, outreach services and community participation). This was followed by more systemic topics, such as quality initiatives, integration of programmes, referral systems, health financing schemes, outsourcing of services and Sector Wide Approaches (SWAp).

In the **supply, distribution and maintenance **thematic area, storage and transport were the main concerns, followed by health equipment, non-health equipment and infrastructure.

### (2) Existing Research Summaries and Syntheses for Priority Topics

Our search yielded 104 citations, of which 51 reviews met the inclusion criteria (see additional file 1: HSS_reviews.xls). A total of 14 (27%) of the included reviews were categorised as being of high methodological quality.

The thematic relevance of the included reviews was high, i.e. all reviews directly addressed one or more of the topics prioritised by countries; geographic relevance was also high in 49% of them. However, only 7 reviews (14%) were rated high for policy relevance. There were only five reviews in total that scored high for both policy relevance and quality (Table 3).

**Table 3:** Cross-tabulation of the number of reviews and overviews according to quality and relevance

	Quality			
			
	High	Moderate/Low	Total	
**Policy relevance**				

High	5	2	**7**	**14%**
Moderate/Low	9	35	**44**	**86%**

**Geographic relevance**				
High	4	21	**25**	**49%**
Moderate/Low	10	16	**26**	**51%**

**Thematic relevance**				
High	7	26	**33**	**65%**
Moderate/Low	7	11	**18**	**35%**

**Total**	**14**	**37**	**51**	**100%**
	**27%**	**73%**	**100%**	

Table 2 also shows the number of high quality reviews or overviews over the total number of reviews for each topic. Many of the reviews were relevant to more than one of the 30 topics. Moreover, there were overlaps in the content of a number of the reviews, i.e. where individual studies were included in more than one review and where individual included reviews were also included in overviews of reviews.

Theme 1: 'health workforce'. For seven of the 10 topics within this theme, at least one review or overview of high quality was identified. No good quality reviews were found for 'numbers of staff per cadre' (appearing in 41% of health systems strengthening proposals), 'performance management systems' (14% of proposals) and 'gender balance of staffing' (7% of proposals).

Theme 2: 'organisation and management'. Eight of the 14 topics had not a single high quality review, and four of those had no reviews or overviews, regardless of their quality: 'financial management procedures' (in one third of the health systems strengthening proposals), 'performance contracts within the public sector' (6 proposals), 'external funding/SWAp' (3 proposals) and 'oversight of the public sector' (2 proposals). The two topics that appeared most frequently in the proposals ('health information' and 'training in management and budgeting') were among those without any high quality review.

Theme 3: 'Supply, distribution and maintenance'. Although each of the six topics in this theme area featured in a high proportion of health systems strengthening proposals (range: 32% to 73%), no relevant reviews of high or moderate quality were found for any of them.

Overall, good quality reviews were identified for four of the topics that appeared in more than half of proposals ('development of staff skills to deliver services', 'increased knowledge, awareness and community empowerment', 'strengthening of supervision' and 'outreach service delivery').

### (4) Use of local evidence

Finally, in matching the availability of nation-wide surveys with their use in the health systems strengthening proposals, we found that in 39 of the 44 countries that submitted proposals there had been a nation-wide household surveys (DHS or MICS) that could have been used to support descriptions of the immunisation status of the population and/or specific barriers to immunisation. However, only 12 of those 39 countries (31%) used survey findings to support their requests (of these, 11 made reference to surveys conducted in the three years prior to proposal submission and one cited an older survey). See Table 4.

**Table 4:** Distribution of health systems strengthening proposals according to the availability and citation of a nation-wide survey

	Number of HSS proposals:		
		
Survey availability:	not citing a survey	citing a survey	Total
Not available or too recent	5	NA	**5 (100%)**
In the previous 3 years	16 (59%)	11 (41%)	**27 (100%)**
Older than 3 years	11 (92%)	1 (8%)	**12 (100%)**

	**32**	**12**	**44**

NA: not applicable.

## Discussion

### Countries' selection of HSS interventions

The World Health Organization has adopted a health systems framework[11] which is increasingly used by stakeholders to analyse health systems issues and to articulate health systems strengthening initiatives[15]. The framework comprises six building blocks: service delivery; health workforce; information; medical products, vaccines and technologies; finances; and leadership and governance. Examining the 30 topics in the proposals from this perspective, we found the topics that appeared most frequently were related to the health workforce, information systems and service delivery building blocks, while topics related to 'leadership and governance' were, in contrast, absent. Most of the proposals were concerned with the operational aspects of the system (e.g. logistic support for supervision, training) rather than with structural changes.

A few topics were present in a great majority of health systems strengthening proposals: 'development of staff skills', 'health information', 'training in management and planning', and 'supplies management'. Research summaries addressing these topics were in general of moderate or poor quality and in only a few cases were they relevant (as defined in the methods section, above). One of the most striking examples is the topic most frequently included in the health systems strengthening proposals we studied: 'health information systems', a topic that appeared in 39 proposals, but for which no reviews of at least moderate quality could be found. Despite the increasing importance of health information systems in the context of health systems strengthening[16], reviews or overviews on health information were difficult to find, perhaps because effectiveness studies using experimental designs may not always be feasible or may not capture critical implementation issues. For example, a well functioning health information system requires not only the appropriate technology and logistics that could be evaluated using experimental or quasi-experimental designs, but also political commitment in the use of information. This would support calls for undertaking operations research to document the strategies used to implement interventions on health information systems[17].

Similarly, no relevant reviews of high or moderate quality were found for any of the 'supply, distribution and maintenance' topics despite the fact that these topics appear in a good number of proposals. In this case, it could be argued that the available research is minimal because it is simply unnecessary. In other words, since a functioning health system cannot lack any of these elements, they are 'no-brainers' that do not require research. On the other hand, some would argue that research could play an important role in determining the most appropriate technologies for particular contexts or the most efficient ways to deliver them. Such information, which might be found in manuals rather than in peer-reviewed publications, would not be retrievable from the sources searched for this study. This would again support the case for "implementation research" to bridge this gap.

An area for which the amount of evidence appears high relative to the priority attached to it by countries is the 'health workforce structure' topic, which includes 'revising the roles of cadres' and 'creating or abolishing cadres'. It has been argued that human resources for health has been neglected by global health initiatives[16] and it may be that countries responded to that perception by downplaying such needs in their proposals to GAVI. It is also possible that countries had already implemented some of the interventions relevant to this topic and hence did not need GAVI health systems strengthening support in this area. Our analysis, however, was unable to determine either the extent to which such changes to health workforce structures might already have taken place, or the extent to which any such changes were informed by evidence.

We recognise, though, that topics found in health systems strengthening proposals could be influenced by factors other than their importance to the countries, such as the complexity of the topics or interventions to address them, or the amount of funding requested for different kinds of interventions from other funding bodies.

### Relevance of available evidence from research summaries

The utility of evidence depends not only on its quality but also on its relevance. The high frequency of reviews of high geographic and thematic relevance reflected the focus of the search strategy, which included terms related to the main topics and geographical scope of health systems strengthening proposals.

Evidence to support health systems strengthening strategies is still extremely weak[9,18]. Several reasons may explain the lack of policy relevant research summaries for health systems: first, research on health systems interventions with internal and external validity is difficult to undertake and the methods to conduct this type of research are not well established. Health systems research is highly contextual. Second, health systems research may be driven by feasibility criteria and researchers' interests rather than by the needs of policy-makers or managers.

This analysis highlights the need for more and better research on health systems and perhaps also a need for greater capacity to produce high-quality evidence on health systems-level interventions[19]. At the same time, there may also be a case for strengthening the ability of managers to demand, appraise and utilise evidence, including evidence that is readily available. Nation-wide household surveys were scarcely cited in the health systems strengthening proposals even though they provide data not only on immunisation coverage but also on household and system characteristics that may be related to the uptake of vaccination.

## Conclusions

Our study found a relatively few high quality research syntheses on health systems interventions of relevance to LMICs, which is consistent with other authors' assertions about the paucity of health systems research[18]. In particular, relevant reviews were not available for many of the interventions prioritised by GAVI-eligible countries to strengthen their health systems.

Given the growing sense of urgency concerning health systems strengthening and the increasing willingness of global health initiatives to provide a common platform for funding health systems strengthening[20], our findings support (a) calls to improve the pool of research evidence on health systems strengthening interventions; (b) the recommendation that global health initiatives not only give more direction and guidance to countries regarding the use of existing evidence from surveys, systematic reviews or other sources, but also encourage them to include more structural health systems interventions in their funding requests.

## Annex 1: Databases and search strategy

Databases used: The Cochrane Library, MedLine and EmBase. While searching MedLine, sidebars showing related items were also checked so that any documents that appeared relevant could be obtained.

### Theme 1: health workforce, distribution and motivation

(developing countries OR low-income countries OR middle-income countries OR Africa OR Asia OR Latin America) AND (health system OR health services) AND (human resources OR health workforce OR health workers OR staff OR personnel)

with various combinations of the following:

cadre

immunisation skills

immunization skills

IMCI skills

MCH skills

primary health care

geographical distribution

gender balance

performance management system

supportive supervision

performance incentives

performance based funding

performance contract

### Theme 2: organisation and management of health services

(developing countries OR low-income countries OR middle-income countries OR Africa OR Asia OR Latin America) AND (health system OR health services)

with various combinations of the following:

demand

access

utilisation

utilization

community participation

community mobilisation

community mobilization

management

needs assessment

planning

setting objectives

setting targets

budget

resource allocation

primary health care

integration

immunisation

immunization

outreach

campaign

referral

financing

financial management

skills

procedures

training

performance contract

performance agreement

service agreement

contracting

outsourcing

quality control

quality assurance

information system

monitoring

evaluation

governance

stewardship

accountability

### Theme 3: supply, distribution and maintenance systems for drugs, equipment, infrastructure

(developing countries OR low-income countries OR middle-income countries OR Africa OR Asia OR Latin America) AND (health system OR health services)

with various combinations of the following:

physical access

geographical access

infrastructure

facilities

buildings

equipment

health equipment

medical equipment

commodities

supplies

storage

transport

logistics

## Competing interests

The authors declare that they have no competing interests.

## Authors' contributions

XBC conceived and designed the study. MK performed the searches and with PG assessed the quality and relevance of systematic reviews. XBC, MK and PG contributed to the manuscript. All authors read and approved the final manuscript.

## Pre-publication history

The pre-publication history for this paper can be accessed here:

http://www.biomedcentral.com/1471-2458/11/449/prepub

## Supplementary Material

Additional file 1 **List of systematic reviews included in this study**. References and main characteristics of systematic reviews. Click here for file
